# Exercise Training Promotes Functional Recovery after Spinal Cord Injury

**DOI:** 10.1155/2016/4039580

**Published:** 2016-12-06

**Authors:** Juanjuan Fu, Hongxing Wang, Lingxiao Deng, Jianan Li

**Affiliations:** ^1^Department of Rehabilitation Medicine of 1st Affiliated Hospital of Nanjing Medical University, School of Rehabilitation Medicine of Nanjing Medical University, Nanjing, China; ^2^Binzhou Medical University, Yantai, China; ^3^Department of Neurological Surgery, Spinal Cord and Brain Injury Research Group, Stark Neurosciences Research Institute, Indiana University, Indianapolis, IN, USA

## Abstract

The exercise training is an effective therapy for spinal cord injury which has been applied to clinic. Traditionally, the exercise training has been considered to improve spinal cord function only through enhancement, compensation, and replacement of the remaining function of nerve and muscle. Recently, accumulating evidences indicated that exercise training can improve the function in different levels from end-effector organ such as skeletal muscle to cerebral cortex through reshaping skeletal muscle structure and muscle fiber type, regulating physiological and metabolic function of motor neurons in the spinal cord and remodeling function of the cerebral cortex. We compiled published data collected in different animal models and clinical studies into a succinct review of the current state of knowledge.

## 1. Introduction

Spinal cord injury (SCI) refers to a series of spinal injuries caused directly or indirectly by external factors. Depending on the segment affected by the injury, symptoms can range from motor and sensory dysfunction, muscle dystonia, and appearance of pathological reflexes. Primary SCI refers to the injury caused by the external forces acting directly or indirectly on the spinal cord. Secondary SCI refers to further damage caused by spinal cord compression, generated by edema, hematoma, compressive fractures, and broken intervertebral disc tissue. Spinal cord injury is characterized by high morbidity, high cost, and young patient age and it often leads to severe permanent disability. SCI not only affects the quality of patients' lives, but it also adds a burden to the family and the society. The latest statistics showed that the global incidence of spinal cord injury is about 236–1009/million people [1]. In USA, about 250,000 people suffer from varying degrees of SCI each year, with an annual rate of up to 28–50/million. Currently, there is a lack of effective clinical therapy to restore nerve function after SCI [2, 3]. Instead, exercise has become the most important quantifiable means for functional recovery [4, 5], and its mechanism has been studied by both clinical doctors and basic researchers. Here, we review recent researches on the mechanisms by which exercise training promotes functional recovery after SCI. Exercise not only directly strengthens paralyzed muscles and promotes motor function recovery but is also promoting brain remodeling, improves spinal microenvironment, and protects damaged distal motoneuron functions, at multiple levels and through various channels, thereby promoting functional recovery.

## 2. Effect of Exercise Training on Cerebral Cortex

In vitro studies have demonstrated that exercise can induce changes in the local neural circuitry, suggesting that afferent activity can activate cortical cells and promote nerve function remodeling [6]. Research on cortical reorganization after peripheral nerve injury also supports the idea of cerebral cortex plasticity [7]. Synaptic contact immaturity is implied by the following observations: the length increase and density decrease of dendritic spines within 7 days in adult rats with the semisection but restoring to normal after 28 days [8]. This lays the foundation for exercise training promoting cortical reorganization after spinal cord injury.

It was shown that exercise after SCI could improve functional prognosis and induce cerebral cortex recombination in the somatic region. These observations have been recorded in both animal experiments and clinical studies. For example, rats exercises after SCI have shown a higher spontaneous firing rate of cortical neurons and enhanced forelimb sensory and sensorimotor stimulation (i.e., the forelimb motion projection area has been expanded to the lower limbs) [9]. In clinical studies, functional magnetic resonance imaging (fMRI) in patients with cervical spinal cord injury has shown that functional improvement after exercise is related to the degree of activation of the motor cortex [10]. Studies using transcranial magnetic stimulation and electroencephalogram (EEG) recordings have further confirmed changes in the cortical sensorimotor area [11]. Compared with healthy subjects, the sensorimotor cortex area associated with the muscle tissue above the damaged region is expanded in SCI patients. Positron Emission Computed Tomography (PET) studies have shown that, for SCI patients, wrist strength exercises increase the activation of the representative area of the contralateral upper limb motor cortex [12]. In addition, case studies have shown that, after complete C6 spinal cord injury, hand exercise can promote functional improvement and increase the representation of the hand muscles in the cerebral cortex [13]. Thus, after SCI, the functional remodeling of the cerebral cortex occurs to promote functional recovery [14].

Moreover, exercise can affect post-SCI remodeling of the brain function, through generation of systemic changes, such as improving blood circulation and neuroendocrine regulation and reducing spasticity [15]. Studies have confirmed that in animals with passive exercise training after spinal cord transection injury, the plasticity related neurotrophic factor, adenylate cyclase type 1 (ADCY1), and brain-derived neurotrophic factor (BNDF) increase in the somatosensory cortex, at levels significantly higher than in animals without training [16]. BNDF is important for neuronal growth and differentiation, and ADCY1 is important for establishment of long-term synaptic plasticity [17]. It was suggested that exercise training could promote brain function remodeling by inducing BDNF expression [18]. Graziano et al. found that, in animals with cycling training after thoracic spinal cord transection, tactile stimulation of the hind paw induced a neural response remapped to the cortical regions of front paws and forelegs under deep anesthesia [16]. Such training also improved the neurological cortical reorganization corresponding to the lower limbs, despite the interruption of afferent input from these limbs [16]. Active exercise can also increase the complexity dendrites in the dentate gyrus and the density of dendritic spines in rats [3], although the functional significance of these changes is not really clear. Similar studies have demonstrated that treadmill training can promote axonal growth [19, 20] and lesion proximal collateral sprouting and increase synaptic establishment [21]. Other similar findings in mature rat hippocampus have also shown that long-term treadmill training can increase the number of astrocytes and neural stem cells in the lower granular cell layer of the dentate gyrus. Exercise stimulates the proliferation of endogenous neural stem cells and generates neurotrophic factors, such as BNDF, which in turn regulate neural plasticity and improve motor function [22, 23]. Some studies have found that early exercise training after the corticospinal motor system injury can restore the contact of corticospinal tract (CST) and the movement projection of primary motor cortex (M1), thus increasing the number of cholinergic intermediate neurons in the ipsilateral and contralateral spinal cord and reducing the physical control disorder [24]. Therefore, passive exercise training of the areas below the spinal cord injury level can promote functional reorganization of the cortex.

Although brain function remodeling is an important mechanism for functional recovery after SCI, excessive function remodeling can result in pathological consequences such as illusion of limb sensation [25] and neuropathic pain [26]. Therefore, further studies and a deeper understanding of the mechanisms of cortical remodeling are necessary in order to adopt the best strategy after the interruption of sensory afferent pathways [27].

## 3. Effect of Exercise Training on the Structure and Function of Spinal Cord

After SCI, the distal neuron pathways undergo a wide range of chemical, electrophysiological, and structural changes, which result in spontaneous neurological remodeling [28]. The effects of exercise training on the structure and function of the spinal cord post-SCI include reconstruction of the neuronal structure; cellular proliferation and differentiation; activation of the metabolism and expression of neuronal substances and neurotrophic factors; and regulation of the cellular electrophysiological function.

## 4. Effect on Neuronal Structure

Experimental studies demonstrate that, after spinal cord injury, the length and density of the distal motor neuron dendrites and the overall neuron cell size are reduced, suggesting that SCI can cause secondary damage to injured distal motor neurons. Studies have even shown that, after thoracic SCI in rats, exercise training can increase the axonal length of the soleus and tibialis anterior motor neurons, within the lumbar spinal cord. Research using synaptophysin immunohistochemistry has shown that treadmill training can significantly increase the formation of the lumbar spinal synapses [3]. Similarly, stepping training after transection of new born rat spinal cord can cause significant pathway changes and increase motor neuron synapse activation by stimulating primary afferent fibers or white matter tracts [29]. The expression of synaptophysin and PSD-95 in the area surrounding the ventral horn of the spinal motor neurons is significantly higher in rats training on the treadmill than in the untrained group [30]. Exercise training can activate the motor neuron N-methyl-D-aspartate (NMDA) receptor [31], by increasing the expression of BNDF and TrkB (tyrosine kinase gene) in spinal cord [32]. NMDA receptors further regulate neuronal survival, dendritic structure, synaptic plasticity, and neuronal circuits. Active training can also increase the expression of neurotrophic factors and nestin-GFP around the ependymal area. This promotes ependymal cell proliferation and differentiation into neural precursor cells (NPC) and further into oligodendrocytes and astrocytes [33], resulting in nerve regeneration and improved functional recovery.

Oligodendrocytes play a key role in leading and efficient signal transmission, and in maintaining and protecting a normal neuronal function. The immature oligodendrocytes markers, transferrin and cyclic nucleotide phosphohydrolase (CNP), increased significantly after seven days of active training [34]. The current consensus is that exercise can induce the neurotrophic factor BNDF, insulin-like growth factor I (IGF-I), and vascular endothelial growth factor (VEGF), to promote spinal oligodendrocyte regeneration [35]. Active training can also elevate the levels of glial fibrillary acidic protein (GFAP), regulate the astrocytes aggregation, and promote astrocytes maturation and differentiation [36].

## 5. Effect on Cell Biochemistry/Metabolism

In rats with treadmill training, the nucleolar area of motoneurons increases and becomes surrounded by basophilic granules. Also, the staining intensity of glucose-6-phosphate dehydrogenase increases, indicating a boost in protein synthesis [37]. After training, motoneurons can transport more axonal protein, through either forward or reverse transport, and thus improve the overall adaptability of the motor units. For example, synaptic protein SNAP25 links synaptic vesicles and presynaptic membrane and motor neuron axons transport synaptic proteins SNAP25 with high selectivity after training [38]. Other proteins, such as the enzyme malate dehydrogenase and the trophic factor calcitonin gene-related peptide, are present in higher amounts in the motoneurons. Post-SCI cycling training can also raise the amount of phosphocreatine-S6 (P-S6) expressed by intermediate neurons and cause dendrites branching of motor neurons [39].

## 6. Effect on Electrophysiological Properties of Motor Neurons

Exercise training can alter the electrophysiological properties of transacted spinal motoneurons, such as the hyperpolarization of resting membrane potential and voltage threshold, the speed increase of action potential, and the increase of after-hyperpolarization potential amplitude of action potential. Studies have shown that depolarization of resting membrane potential (RMP) and spike trigger level (STL) occurs four weeks after complete transection of the thoracic spinal cord [40]. After training, the RMP can become hyperpolarized [41], thus altering the inhibition of the rubrospinal tract on stepping and promoting functional recovery [29]. Stepping training can enhance muscle spindle afferent signals [42, 43], promote aspartate NMDA receptors functioning [44], and increase the amplitude of the incoming signal of the motoneuron synapsis, causing an after-hyperpolarization in the action potential of motoneurons. In addition, the ventrolateral spinal cord white matter (VLF) can also induce changes in the electrophysiological activity of motoneurons.

In rats with SCI, a change in the after-hyperpolarization potential (AHPd) affects the rhythm, intensity, and duration of interneuron activity which affects the stepping function [45]. Passive exercise can enhance the magnitude of excitatory postsynaptic potentials (EPSP), increase the number of motoneurons that accept incoming signal, steer AHPd towards normal level, and restore normal stepping [46]. Ultra-microstructural analysis has shown that, after spinal cord transection, exercise training can increase the magnitude of gastrocnemius motor neuron (MNs) excitatory postsynaptic potential (EPSP) but has no significant effect on the inhibitory postsynaptic potential (IPSP).

Spinal cord transection injury results in impaired stepping ability, causes reduced incoming signal from distal motoneuron synapses, enhances inhibitory effects, and thereby inhibits *α*- and *γ*-MNs activity. Studies suggest that this may be related to the significantly more numerous inhibitory F-type enlarged terminals than in the excitatory S-type enlarged terminals. It could also be due to the structural changes occurring in the parallel C-type and M-type enlarged terminals of *γ*-MNs cell bodies. Exercise training can maintain a normal ratio between the excitatory S-type and the inhibitory F-type enlarged terminals of *γ*-MNs and *α*-MNs [47], as such maintaining the ratio between excitatory and inhibitory signals to improve stepping function.

## 7. Effect of Exercise Training on the Structure and Function of Skeletal Muscles

After SCI, paralyzed muscles exhibit decreased fiber diameter, reduced voluntary contraction force, decreased metabolism, delayed conversion of slow-twitch to fast-twitch fibers, and a cross-sectional area comprised mainly of type I fibers. Currently, it is believed that skeletal muscle atrophy is characterized by lost [48, 49] or apoptotic muscle fiber nuclei [50], suggesting that reduced myoglobin nuclei number leads to “nuclear apoptosis.”

The lost function characterizing muscle atrophy can be restored by several methods, primarily by inducing IGF-1 [51], Pax7, and other molecules that promote myogenic cells (satellite cells) activation, proliferation, and differentiation and participating in muscle fibers (muscle cells) repair [52]. Studies have shown an increase in the soleus IGF-1 protein levels after spinal cord injury. After treadmill training, soleus IGF-1 shows additional increase, to activate proliferation and differentiation of satellite cells [53], and increase in the muscle fiber numbers. This implies that exercise training after SCI can enhance satellite cell activity and promote muscle fiber formation. Endurance training seems to increase terminal branching of nerves at the neuromuscular junction [54], although results are ambiguous [55].

Studies have shown that in cats with SCI the expression the levels of myosin heavy chain (MHC) in the soleus can be restored after stepping training. After one week of weight-bearing stepping training after SCI, the wet weight of the plantaris, medial/lateral gastrocnemius, soleus, and tibialis anterior muscles is significantly reduced. After three weeks of training, twitching and tonic tension peaks in the soleus muscle decrease significantly and the MHC expression in the extensor digitorum longus IIx increases. By 10 weeks, the muscle wet weight, contractile properties, and MHC levels returne to baseline levels, except for LG/MG atrophy [56].

## 8. The Effect of Combinatorial Strategy with Exercise

Although exercise training can improve neurological and skeletal muscle function by modulating the multilevel structures and function of the cerebral cortex, spinal cord, and skeletal muscle following spinal cord injury (SCI), current evidence suggests that exercise training has limited efficacy in improving motor function after SCI in rodents or cats [57–60]. Such inadequate effects are believed to be attributed to insufficient neurotrophic factor production induced by training. Therefore, in addition to exercise, more and more combinatory strategies have been investigated. Many studies have shown that combined therapy can significantly promote the recovery after SCI and relieve spasticity in rats compared to single treatments alone [61]. Current strategies focus on combinatorial effects of hematopoietic stem cells, neurotrophic factors, drugs, and electrical or magnetic stimulation. Tashiro et al. showed that neural stem cell transplantation combined with treadmill training significantly improved spinal cord pathway conduction and increased central pattern generator activity, resulting in significantly improved motor function [62]. Dental pulp stem cell transplantation not only promoted motor function recovery but also significantly reduced lesion cavity and glial scar formation [60]. Trophic support appears to be the key to effective combination therapy. The secretion of neurotrophic factors can be stimulated in the injured spinal cord by neural stem cell transplantation or exercise training [20, 63, 64]. Combining these two therapies can significantly increase neurotrophic factor secretion. Direct neurotrophic factor application combined with exercise training can also promote functional recovery following SCI [65]. Han et al. combined Glial cell line-derived neurotrophic factor (GDNF) with early rehabilitation significantly reduced pathological changes and motor dysfunction in patients with SCI [61]. Other neurotrophic factors such as Brain-derived neurotrophic factor (BDNF) and Neurotrophin-3 (NT-3) can also significantly increase BBB scores, indicating improved functional recovery [66]. The above studies suggest that trophic support from combinatorial treatment is an effective intervention to improve motor recovery after spinal cord injury.

In addition, many studies have shown that electrical stimulation or magnetic stimulation combined with exercise training can influence motor function in SCI patients [67]. Petrosyan et al. showed that spinal cord electrical stimulation combined with exercise training can induce sustained enhancement of synaptic transmission, thereby improving lumbar anatomical plasticity to promote motor function recovery [59]. Chao et al. demonstrated that functional electrical stimulation combined with treadmill training could activate intraspinal circuits to improve gait control [68]. After SCI, load-bearing training combined with functional electrical stimulation can activate ankle flexion to prevent swing phase drag, also reduced swing phase time and improved limb coordination [69, 70]. Sensory stimulation of the tongue combined with specific training significantly improved the balance and gait of patients with incomplete SCI [71]. There are also reports that repetitive transcranial magnetic stimulation combined with plate training can significantly reduce lower limb stiffness in patients with SCI [72]. Therefore, the combination of electrical or magnetic stimulation and exercise training could significantly improve motor function and maximize functional recovery, which provides a new perspective for clinical rehabilitation.

The effects of drug therapy combined with exercise training have also been reported. Intraperitoneal injection of fluoxetine combined with exercise training significantly increased BDNF in the hippocampus which promoted nerve regeneration and BBB score [73, 74]. The combined use of meta-chlorophenylpiperazine (mCPP) and quipazine in SCI not only improved BBB scores but also improved weight-bearing walking [66]. The implantation of polypyrrole/iodine (PPy/I) can protect nerve tissue and promote functional recovery [75]. If combined with treadmill exercise training, this effect is more significant [2]. Alluin et al. showed that combination therapy of chondroitinase ABC, neurotrophic factors, and exercise training not only enhanced active motor function recovery by enhancing neuroanatomical plasticity of the descending tracts (corticospinal tract and 5-HT pathway) but also significantly reduced the astrocyte proliferation and inflammation around lesions [76]. Transplantation of Schwann cells and olfactory ensheathing cells in the spinal cord of cats combined with chondroitinase ABC treatment significantly improved motor function [77]. Drug therapy combined with exercise training is more favorable in the treatment of SCI, suggesting that adjuvant drug treatment may have a better prognosis in SCI patients in the clinic.

In conclusion, exercise training can induce structural and functional changes in the cerebral cortex, spinal cord, and skeletal muscles, thus improving neural and muscular function following spinal cord injury. Exercise appears to promote nerve regeneration with functional restoration, to induce corticospinal pathway connectivity [36], to maintain the functional status of spinal cord neurons, to activate skeletal muscle satellite cells, and to promote muscle fiber regeneration. Exercise training combined with other treatments in SCI is the future direction with the most promise. More research is needed to optimize the specific training parameters, such as intensity, duration, frequency, and so forth. Also, the effect of exercise on SCI secondary complications (e.g., chronic pain, bladder and gastrointestinal dysfunction, muscle mass loss, osteoporosis, pressure ulcers, joint and muscle pain, fatigue, sleep problems, depression, and temperature control loss) is rarely discussed in literature and needs further exploration.
